# Two new jumping spider species of the *Habronattus clypeatus* group (Araneae, Salticidae, Harmochirina)

**DOI:** 10.3897/zookeys.625.9891

**Published:** 2016-10-19

**Authors:** Wayne P. Maddison, David R. Maddison

**Affiliations:** 1 Beaty Biodiversity Museum and Departments of Zoology and Botany, University of British Columbia, 6270 University Boulevard, Vancouver, British Columbia, V6T 1Z4, Canada; 2 Department of Integrative Biology, Oregon State University, Corvallis, OR 97331, USA

**Keywords:** Araneae, Salticidae, Salticinae, Plexippini, Harmochirina, jumping spider

## Abstract

Two species of the *Habronattus clypeatus* species group are described, *Habronattus arcalorus* **sp. n.** from Texas and Colorado, and *Habronattus gilaensis* **sp. n.** from New Mexico. *Habronattus arcalorus* males have extravagant ornamentation: a green first leg with an unusually dense lateral fringe of orange and white hairs, and a large grey triangular patella on the third leg with blue-white scales nearby. *Habronattus gilaensis* males are considerably more muted, lacking ornamentation on the third leg’s patella and tibia. Photographs of living specimens are given, as well as notes on habitat.

## Introduction

The genus *Habronattus* F.O. Pickard-Cambridge, 1901 includes jumping spiders whose males have remarkably complex courtship displays, especially the members of a large clade with modified first and third legs (Peckham and Peckham 1890; Griswold 1987; Elias et al. 2003, 2012). Within this clade, the first leg of most males is laterally fringed with modified setae, and the third femur, patella and tibia have several modifications of apophyses, swellings, tufts, and colours. This clade contains 38 described species (Griswold 1987) divided into three species groups, of which the *clypeatus* species group is restricted to the southwestern United States and Mexico. We here describe two new species of the *clypeatus* group, one of which has some of the most exaggerated courtship ornamentation known in *Habronattus*. These two species were referred to in molecular phylogenetic and chromosome studies by the names “*Habronattus* sp. (CNCTY)” and “Habronattus cf. dossenus” (Maddison and Hedin 2003; Maddison and Leduc-Robert 2013), and are the current focus of continuing behavioural and molecular studies.

## Methods

Preserved specimens were examined under both dissecting microscopes and a compound microscope with reflected light. Drawings were made with a drawing tube on a Nikon ME600L compound microscope. All specimens are deposited in the Spencer Entomological Museum of the University of British Columbia
(UBC-SEM), except for paratypes deposited (as noted) in the AMNH (American Museum of Natural History), MCZ (Museum of Comparative Zoology, Harvard University) or OSAC (Oregon State Arthropod Collection, Oregon State University, Corvallis).

Terms are used in standard fashion for Araneae. The descriptions were written with primary reference to the focal specimen indicated, which was used for measurements and carefully checked for details, but they apply as far as known to the other specimens examined. All measurements are given in millimeters. Carapace length was measured from the base of the anterior median eyes (AME) not including the lenses to the rear margin of the carapace medially; abdomen length to the end of the anal tubercle. Positions on the bulb of the male palp (left, ventral view) are described using hours of an analog clock’s face. The following abbreviations are used: ALE, anterior lateral eyes; PLE, posterior lateral eyes; PME, posterior median eyes (the “small eyes”).

### The *Habronattus clypeatus* species group

**Figures 1–9.**
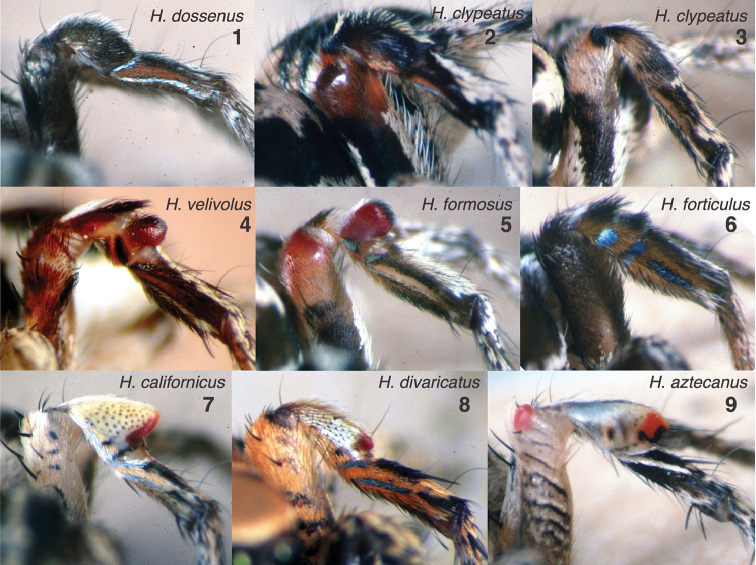
Third legs of males of described species in the *Habronattus clypeatus* species group. Left legs shown, except Figs 1, 2, 7, 9 which show digitally-flipped images of right legs. **1** *Habronattus dossenus* Griswold, 1987, from U.S.A.: Arizona: Cochise Co.: Lower Turkey Creek, Chiricahua Mts, 109.42°W, 31.85°N, **2** *Habronattus clypeatus* (Banks, 1895) (montane form), from U.S.A.: Arizona: Jack’s Canyon near Flagstaff **3** *Habronattus clypeatus* (desert form), from U.S.A.: Arizona: Pima Co.: Santa Catalina Mts., Molino Basin, Prison Camp road **4** *Habronattus velivolus* Griswold, 1987, from México: Jalisco: Laguna Sayula, 19.9963°N, 103.5522°W **5** *Habronattus formosus* (Banks, 1906), from U.S.A.: California: Inyo Co.: Bishp **6** *Habronattus forticulus* (Gertsch & Mulaik, 1936), from U.S.A.: Texas: Travis Co., Austin, 30.25°N, 97.71°W **7** *Habronattus californicus* (Banks, 1904), from U.S.A.: California: San Diego Co.: Oceanside, 33.27°N, 117.44°W **8** *Habronattus divaricatus* (Banks, 1898), from México: Baja California Sur: near La Laguna trailhead, San Juan del Aserradero **9** *Habronattus aztecanus* (Banks, 1898), from México: Nayarit: San Blas. All photos credit W. P. Maddison.

The eight described species of this group can be distinguished from one another by their male third legs (Figs 1–9) as well as other features. The group is distinctive for having the male clypeus with vertical-oblique dark bands descending from the AME, the basal white band of the abdomen divided by one or two longitudinal medial dark bands, and (usually) a medial longitudinal dark band on the underside of the abdomen. The retrolateral tibial apophysis appears as a hook in ventral view. In addition, in living males of the *clypeatus* group a pattern of dark spots is visible when the observer looks into the AMEs, consisting of an array of dark pigment patches (see, for example, this video: https://www.youtube.com/watch?v=Dq5ky7vjPYo). It is possible that all *Habronattus* have such a pigmentation pattern in the eyes, but that it has been observed only in the *clypeatus* group because the broad depigmented areas on the thorax let in light.

Species boundaries in the group are not entirely clear. *Habronattus clypeatus* (Banks, 1895) has two forms, a montane form in most of its range, dark and with the third femur swollen and orange terminally (Fig. 2), and a smaller paler form from the deserts of southern Arizona whose third femur is less modified (Fig. 3). The desert form appears to intergrade with *Habronattus formosus* (Banks, 1906) (Fig. 5), with the third leg’s patella becoming more red and swollen moving west from Phoenix to Yuma. Most recognized species are parapatric or allopatric, but there is at least one case of sympatry: *Habronattus dossenus* Griswold, 1987 and *Habronattus clypeatus* in southern Arizona, where they can co-occur in the same small habitat. In such situations, apparent hybrids – male specimens that blend the ornaments of both species – can be found occasionally. The two species described here are distinctive, without any known intergradation with other forms.

## Results

### Habronattus arcalorus

Taxon classificationAnimaliaAraneaeSalticidae

Maddison & Maddison sp. n.

http://zoobank.org/479C8BE5-6F5D-470E-9ECC-2F63175C6159

Figs 10–21

#### Holotype.

Male in UBC-SEM, with labels: “TEXAS: Jeff Davis Co.: Davis Mountains State Park 103.939°W, 30.593°N, 22-23 April 1997 Maddison/Hedin/Hebets WPM#97-001” and “♂W167”.

#### Paratypes

(5♂♂ 3♀♀). 1♂ (specimen W328) 1♀ (specimen W332) in UBC-SEM; 1♂ 1♀ in AMNH; 1♂ 1♀ in MCZ; 2♂ in OSAC; all with same data as holotype.

#### Etymology.

The name is an arbitrary combination of letters, including the syllable “arc”, included to evoke the rainbow of colors in the male ornaments.

#### Diagnosis.

The large triangular patella of the third leg of males is distinctive in salticids. *Habronattus viridipes* (Hentz, 1846) has a triangular patella also (Griswold 1987: figure 99), but it is different from that of *Habronattus arcalorus* in colour (orange-yellow with a black spot, rather than grey) and in having a terminal apophysis. *Habronattus californicus* (Banks, 1904), also of the *clypeatus* group, has a triangular patella (Griswold 1987: figure 107), but the patella is smaller and yellow and red. *Habronattus arcalorus* also differs from *Habronattus californicus* in having green first legs with a much denser fringe. Females of *Habronattus arcalorus* are distinctive from most specimens of other *clypeatus* group species in having a distinct black triangle on the thorax (Figs 19–20).

**Figures 10–21.**
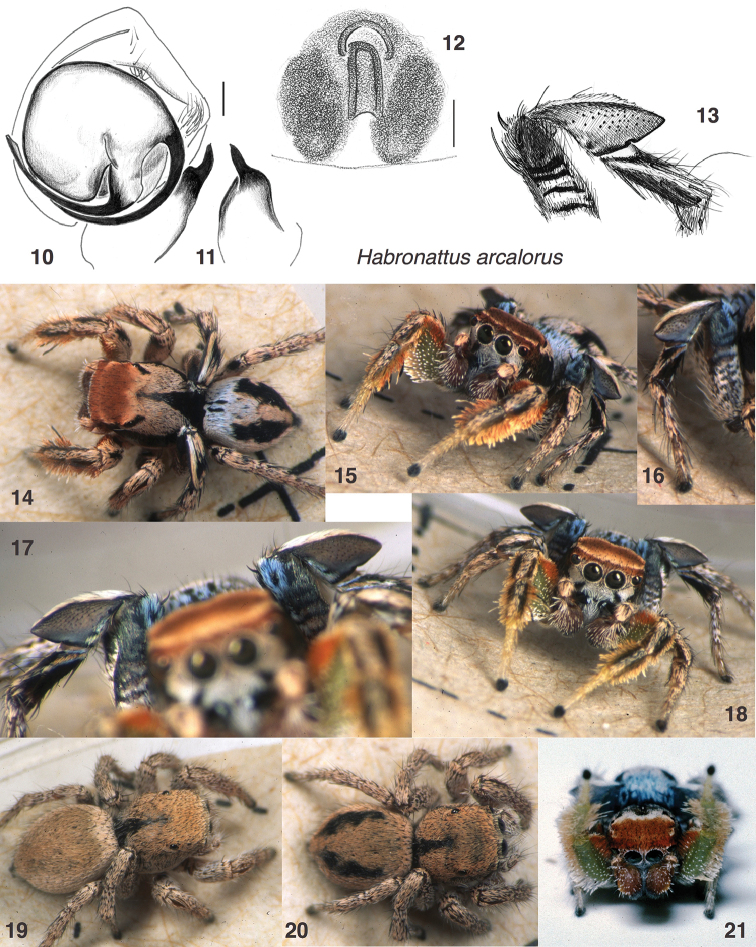
*Habronattus arcalorus* sp. n. All specimens from Davis Mountains, Texas except Fig. **12**, from Cañon City, Colorado. **(10–11** paratype male W328) **10** left palp, ventral view **11** left palp, retrolateral view. **12** epigynum, ventral view, paratype female W332. Scale bar = 0.1 mm. 4 third leg femur, patella and tibia **13–18** living male **19–20** living females. **21** male in courtship pose. All photos credit W. P. Maddison.

#### Note.

This species was referred to by Maddison and Hedin (2003) as *Habronattus* sp. (CNCTY) (and possibly also *Habronattus* sp. (CHIH); see note below on Chihuahuan form). Molecular data suggest that *Habronattus arcalorus* and *Habronattus forticulus* (Gertsch & Mulaik, 1936) are outside the clade containing the bulk of the *clypeatus* group species – *Habronattus aztecanus* (Banks, 1898), *Habronattus californicus* (Banks, 1904), *Habronattus clypeatus*, *Habronattus divaricatus* (Banks, 1898), *Habronattus dossenus*, *Habronattus formosus*, and *Habronattus velivolus* Griswold, 1987. *Habronattus arcalorus* and *Habronattus forticulus* are the easternmost representatives of the *clypeatus* group.

#### Description.

*Male* (focal specimen: holotype, specimen W167). Carapace length 2.3; abdomen length 2.3. Structure of chelicerae, legs, and body typical for *Habronattus* and the *clypeatus* group. Palp (Figs 10, 11) standard for the *clypeatus* group (Griswold 1987 figures 180, 181, 203), with the embolus arising at about 3:00 and the basal portion of the terminal apophysis pointing to 6:00. The tibial apophysis is hooked from ventral view (Fig. 10); in retrolateral view it narrows abruptly before its tip leans ventrally (Fig. 11). First legs (Figs 14, 15, 18, 21): Fringed laterally on the femur, patella, and tibia. Femur prolaterally and ventrally covered with spoon-shaped setae, swollen at the tips. Tibia with two large spatulate macrosetae prolaterally. Third legs (Figs 13, 16, 17): Patella swollen into a large triangle. Colour in life (Figs 14–18, 21): Chelicerae dark, with white setae medially. Palp femur and patella covered with beige scales above; cymbium with scattered white erect setae, especially retrolaterally. First leg dark above, but with light green integument below, and with dense fringes of erect setae prolaterally and retrolaterally on the femur, patella and tibia. These fringes are orange on the femur prolaterally, grading to white elsewhere on femur, patella, and tibia. Spoon-shaped setae on underside of first femur are white at tips. Femur of third leg with transverse white and dark bands (Figs 13, 16) in basal half, blue-white scales in distal half that match similar blue-white scales at front of abdomen (Figs 14–18). Third patella steel-grey with a white fringe above; tibia black with pale band rising obliquely toward the distal and dorsal (Figs 13, 17). Clypeus covered in white setae except for a dark band descending obliquely from each AME. Ocular area rust-coloured, with thin tan band arching between PMEs. Pale thoracic bands wide anteriorly, narrowing to a point at back margin. Abdomen with generous basal band of blue-white scales, broken at the front by two small black lines (Fig. 14). Longitudinal medial band of pale scales on dorsum of abdomen triangular, much wider at front than back. Colour in alcohol: more or less as in life, but with first leg integument yellow-orange, no longer green, and bluish cast of third femur and abdominal basal band not visible.

*Female* (focal specimen: paratype, specimen W332). Carapace length 2.7; abdomen length 2.8. Structure typical for *Habronattus* and the *clypeatus* group. Epigynum (Fig. 12) with long narrow central pocket in front of which is a small semicircular atrium, as is usual for the species group. Colour (Figs 19–20): Uniform beige to tan, with black triangular patch on posterior thorax. Some specimens show two longitudinal dark bands on abdomen (Fig. 20). The clypeus is covered with white scales, but a hint of the dark oblique bands of the male can be seen beneath the white scales. Likewise, there is a hint of the transverse bands on the third femur.

#### Additional material examined.

USA: TEXAS: Jeff Davis Co.: Davis Mountains State Park 103.939°W, 30.593°N, 22–23 April 1997 Maddison/Hedin/Hebets WPM#97-001 (11 ♂♂, 2 ♀♀, 6 subadult ♂♂, 16 other juveniles). TEXAS: Jeff Davis Co.: Davis Mountains, roadside, highway 118 104.098°W, 30.704°N, 23 April 1997 Maddison/Hedin/Hebets WPM#97-003 (2 ♂♂ 1 ♀). COLORADO: Fremont Co., Royal View Campground. ~9 mi W of Cañon City on HWY 50. N38.495° W105.354°. 24 May 1982 D. & W. Maddison WPM#82-108 (10 ♂♂, 6 ♀♀).

#### Chihuahuan form.

**Figures 22–26.**
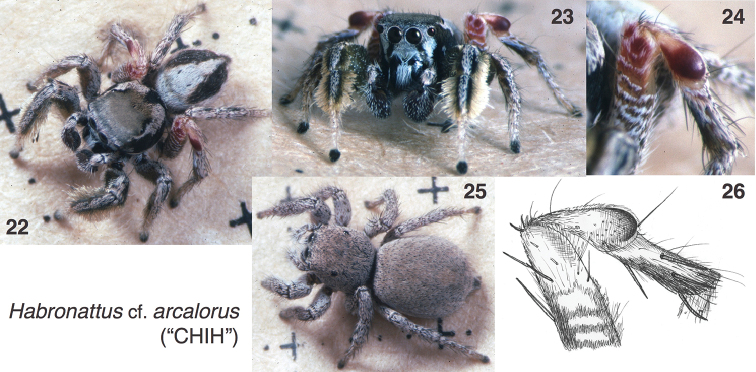
Habronattus cf. arcalorus (“CHIH”). All specimens from near Tomochic, Chihuahua. **22–23** living male. **24** third leg, living male **25** female **26** third leg in alcohol. All photos credit W. P. Maddison.

Specimens of a form that may be either a geographical variant of *Habronattus arcalorus*, or a distinct species, were found in the Tomochic area of Chihuahua, Mexico (Figs 22–26). This is the form referred to by Maddison and Hedin (2003) as “*Habronattus* sp. (CHIH)”. The markings, robust body, and dense fringes on the male first leg match those of *Habronattus arcalorus*, but the third patella is different (Figs 24, 26): smaller, rounded, dark red in the distal half. The tip of the femur is dark red with white bands, not blue-white as in *Habronattus arcalorus*. The front of the abdomen is not bluish, nor does the basal band have two clear dark stripes. *Habronattus arcalorus* and the Chihuahuan form do not group together by mitochondrial data in Maddison and Hedin’s (2003) gene tree, but Maddison and Hedin suggest there is introgression from the *coecatus* group into the Chihuahuan form, and nuclear data are lacking. It seems likely that a diversity of geographic forms exists in satellite ranges of the Sierra Madre Occidental and Sierra Madre Oriental in Chihuahua and Coahuila, yet to be discovered. Until more detailed studies are done, we will avoid giving a new taxon name to the Chihuahan form, and call it Habronattus cf. arcalorus (“CHIH”). The material examined of the Chihuahuan form (all in UBC-SEM) is: MEXICO: CHIHUAHUA: 1 mi E of Tomochic on HWY 16. 14 Mar 1996 Susan Masta (6 ♂♂, 5 ♀♀). CHIHUAHUA: 2.7 mi E of Tomochic on HWY 16. 14 Mar 1996 Susan Masta (2 ♀♀). CHIHUAHUA: 10.1 mi E of Tomochic on HWY 16. 14 Mar 1996 Susan Masta (4 ♂♂, 4 ♀♀). CHIHUAHUA: 15 mi N of Madera road to 40 Casas archaeological zone. 15 Mar 1996 Susan Masta (2 ♂♂, 1 ♀).

#### Natural history.

*Habronattus arcalorus* was found abundantly in both areas in which it was collected. At the Cañon City locality it was found on rocks on a dry hillside with sparse vegetation and pinyon pines (Fig. 33). In the Davis Mountains of Texas it was found on oak leaf litter mixed with grass and rocks in a stream valley, and on rocks mixed with grass in a juniper-pinyon-oak woodland. Fig. 21 shows a male in courtship pose. Maddison and Leduc-Robert (2013) report the karyotype of *Habronattus arcalorus* as 26 acrocentric autosomes plus 2 acrocentric X chromosomes in males, as is typical for salticids.

### Habronattus gilaensis

Taxon classificationAnimaliaAraneaeSalticidae

Maddison & Maddison sp. n.

http://zoobank.org/B708C045-57ED-4286-806E-E96615C6ADF3

Figs 27–32

#### Holotype.

male in UBC-SEM, with labels “USA: New Mexico: Grant Co., Bill Evans Lake, 1400 m. 32.865°N, 108.5784°W, 10 April 1996 D.R. Maddison DRM96.015” and “W123”.

#### Paratypes

(5♂♂ 2♀♀). New Mexico: Grant Co., Gila River, Billings Vista, 32.8137°N, 108.6011°W, 11 August 2005 D.R. Maddison DRM 05.042 (1♂ in each of AMNH, MCZ, OSAC; 1♀ in UBC-SEM); New Mexico: Grant Co., Bill Evans Lake, 1400 m. 32.865°N, 108.5784°W, 10 April 1996 D.R. Maddison DRM96.015 (1♂ in UBC-SEM); New Mexico: Grant Co., route 90 S of Tyrone, 1755 m. 32.702°N, 108.3043°W, 10 April 1996 D.R. Maddison DRM96.016 (1♂ in UBC-SEM); New Mexico: Walnut Creek Road NW of Silver City, 32.8309°N, 108.3326°W, 6 April 2012, C. Wu (1♀ in UBC-SEM, “female 434” of D. Elias laboratory);

#### Etymology.

The name refers to the known distribution of this species, in the region of the Gila River drainage of New Mexico.

#### Diagnosis.

The third leg has an unornamented patella and tibia (Fig. 30), unlike all other members of the *Habronattus clypeatus* group (Figs 1–9). The only other species of the group with green first legs in the male are *Habronattus dossenus* and *Habronattus arcalorus*. From *Habronattus arcalorus*, *Habronattus gilaensis* differs in many details of ornamentation, especially the third leg. *Habronattus gilaensis* is most similar to *Habronattus dossenus*, but differs (in males) in almost complete lack of ornamentation on the third leg, in the paler ocular area, denser and longer fringes on the first leg, denser covering of white setae on the cymbium, and in having the white scales on the side of the carapace broken by a black band extending posteriorly.

**Figures 27–32.**
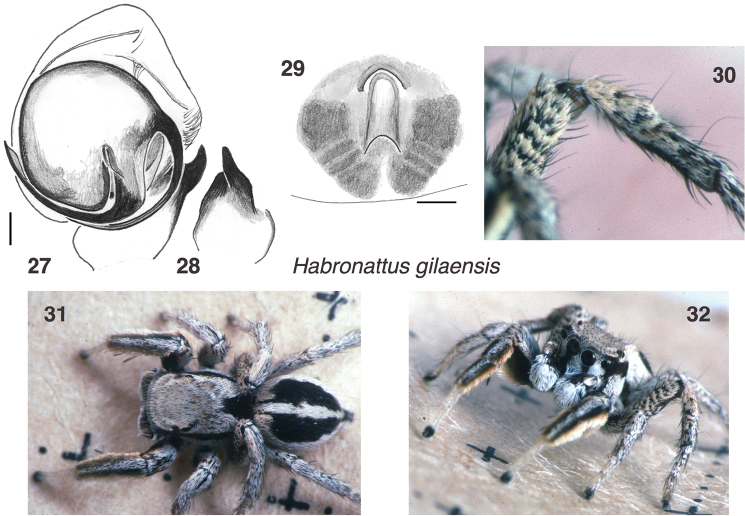
*Habronattus gilaensis*, sp. n. **27–28** male holotype left palp **29** Paratype female epigynum, ventral view **30–32** living male from south of Tyrone, New Mexico (32.702°N 108.3043°W) **30** is anterior lateral view of third leg. All photos credit W. P. Maddison.

#### Note.

This species was referred to as Habronattus cf. dossenus by Maddison and Hedin (2003).

#### Description.

*Male* (focal specimen: holotype, specimen W123). Carapace length 2.2; abdomen length 2.0. Structure of chelicerae, legs, and body typical for *Habronattus* and the *clypeatus* group. Palp (Figs 27–28) standard for the *clypeatus* group, very similar to that of *Habronattus arcalorus*. First legs (Figs 31, 32): Dense fringes of erect setae retrolaterally on the femur, and prolaterally and retrolaterally on the patella and tibia. The setae of these fringes are simple, not swollen as in *Habronattus arcalorus*. Tibia with two large spatulate macrosetae prolaterally. Third legs (Figs 30, 32): Almost as in female, little modified. Colour in life (Figs 31, 32): Chelicerae dark, with white setae medially. Palp femur and patella covered with beige scales above; cymbium fairly densely covered with white setae. First leg dark above, but with green integument below, and fringes tan to white. Femur of third leg with indistinct transverse bands (Figs 30, 32). Clypeus covered in white setae except for a dark band descending obliquely from each AME. Ocular area light brown to tan, with thin tan bands arching between PMEs. Pale thoracic bands wide anteriorly, narrowing to a point at back margin. Abdomen with basal band of white scales broken at the front by two small black lines (Fig. 31). Longitudinal medial band of pale scales on dorsum of abdomen narrow. Medial longitudinal dark band on underside of abdomen. The dark clypeal bands, pale arching band in the ocular area, and broken basal band are also typical for the *clypeatus* group. Colour in alcohol: more or less as in life, but with first leg integument yellow-orange, no longer green.

*Female* (focal specimen: paratype, “female 434”). Carapace length 2.6; abdomen length 3.2. Structure typical for *Habronattus* and the *clypeatus* group. Epigynum (Fig. 29) with long narrow central pocket in front of which is a small semicircular atrium, as is usual for the species group. Colour: uniform beige to tan scales covering body. Clypeus covered with white scales. Third femur with a hint of the transverse bands seen in the male.

#### Additional material examined.

USA: New Mexico: Grant Co., route 90 S of Tyrone, 1755 m. 32.702°N, 108.3043°W. 10 April 1996. D.R. Maddison. DRM96-016 (3 ♂♂). New Mexico: Grant Co., Big Burro Mountains, road from Lordsburg to Silver City. 32.505°N, 108.464°W. 9 August 1997. W. Maddison & M. Hedin. WPM#97-028 (1 ♂).

#### Natural history.

Found on open dry ground, on rocks and leaf litter (Fig. 34).

**Figures 33–34.**
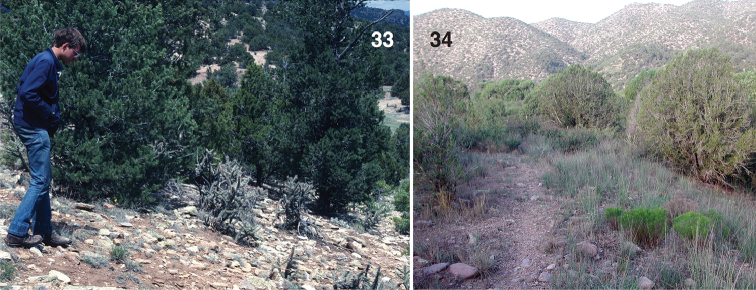
Habitats of *Habronattus arcalorus* and *Habronattus gilaensis*. **33** *Habronattus arcalorus* habitat with second author searching for specimens, USA: Colorado: ~9 mi W of Cañon City, 38.495°N, 105.354°W, 24 May 1982 WPM#82–109 **34** *Habronattus gilaensis* habitat (on ground in foreground), USA: New Mexico: Grant Co., Gila River, Billings Vista, 32.8137°N, 108.6011°W, 11 August 2005 DRM 05.042.

## Supplementary Material

XML Treatment for Habronattus arcalorus

XML Treatment for Habronattus gilaensis
